# Supplementary material for: Characterisation and Expression Analysis of LdSERK1, a Somatic Embryogenesis Gene in Lilium davidii var. unicolor
Source: Plants (Basel). 2024 May 29;13(11):1495. doi: 10.3390/plants13111495 (PMC11174594; doi:10.3390/plants13111495)
Supplement: Supplementary file 1 [file plants-13-01495-s001.zip › plants-2957565-supplementary.pdf]

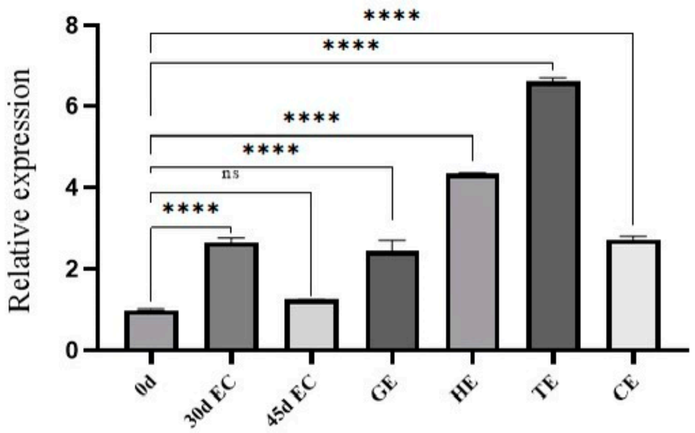

Figure S1. Expression of the *LdSERK1* gene at different times of embryo callus and at different periods of somatic embryo development.

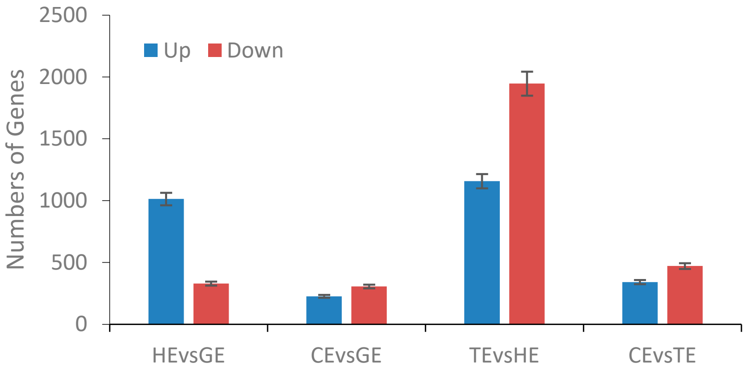

Figure S2. Statistical map of the number of differential genes at different stages of somatic embryo.

Table S1. Kan resistance screening of Lanzhou lily scales

Group	Number of infections	Kan concentration(mg/L)	Browning rate (%)	Scale growth status
A	30	0	0	growing well
B	30	60	13.33%	no obvious browning
C	30	90	30.00	Minor browning
D	30	120	53.33	Severe browning
E	30	150	63.33	Browning too severe

Table S2. Screening of scales for cef resistance of Lanzhou lily scales

Group	Number of infections	Kan concentration(mg/L)	Browning rate (%)	Scale growth status
F	30	0	0	growing well

J	30	300	6.67	no obvious browning
H	30	400	16.67	Minor browning
I	30	500	30.00	Severe browning
J	30	600	46.67	Browning too severe

Table S3. List of gene-specific primers used for gene cloning.

	Gene and primer name	Forward primer (5' to 3')	Reverse primer (5' to 3')	Source
**Gene cloning for pGXT-LdSERK1**	*LdSERK1* (LdF1/LdR1)	ATGGCGGTCGCAGAGCGG	TCGAGCTATCAGGCCCTAGGTGA	This study
**Gene cloning for pCAMBIA1302-LdSERK1**	*LdSERK1* (LdF2/LdR2)	ACCATGGTAGATCTGACTAGTATGGCGGTCGCAGAGCGG	AAGTTCTTCTCCTTTACTAGTCCTAGGGCCTGATAGCTCGA	This study
**Gene cloning for TRV2-LdSERK1**	*LdSERK1* (LdF3/LdR3)	TGAGTAAGGTTACCGGAATTCGAAGTTCATCTCGGTCAGCT	GGGACATGCCCGGGCCTCGAGATAGACAAGCAGACGTTCGG	This study
**RT-PCR for CP**	*CP* (CPF/CPR)	CTAACAGTGCTCTTGGTGTGATT	CAACTCCATGTTCTCTAACGAAGT	This study
**RT-PCR for MP**	*MP* (MPF/MPR)	CGCAGTACAAGGTTGAATACAGT	CTCAATCGTCTTCATCTCCACTT	This study
**qRT-PCR for LdSERK1**	*LdSERK1* (LdF/LdR)	ATATTTCTTTGACGTACCTGCTG	GCCCAAAATATTCTTGTTGCTGA	This study
**EF-1a as an internal reference gene**	*EF-1a(EFF/EFR)*	GCATCACACCTTCTACAACG	GAAGAGCATAACCCTCATAGA	This study
**PCR for Hyp gene**	*Hyp(HypF/HypR)*	TACACAGCCATCGGTCCAGA	TAGGAGGGCGTGGATATGTC	This study
**actin as an internal reference gene**	*Actin (ActinF/ActinR)*	GCTGAGAGTTGATGGTGTGCT	GGATACCCTTTCGCAGATAGAG	This study

LdSERK1 gene cDNA whole sequence: (GenBank: OP752101.1)

>transcript7671/f9p0/2416

ATGGCGGTCGCAGAGCGGGCGTGGTTTCTGATACTGGTTTTGTTGCTTCGGCCAATCGC
TAGGGTTTCGGCCAACACGGAAGGCGATGCATTGCATAGTTTGCGGACCAACTTAAAT
GATCCTAACAACGTACTGCAAAGTTGGGATCCTACTTTGGTCAATCCATGCACATGGTT
CCATGTTACATGTAACAATGATAATAGTGTTATTAGAGTTGATCTTGGTAATGCAGCTTT
ATCTGGTTCACTGGTTCCTCAACTTGGTCAGCTGAAAAATTTGCAATACCTGGAGCTTT
ACAGTAACGACATAAGTGGGTTTATTCCCAATGAACTTGGTAATCTGACAAACCTGGTG
AGTTTGGATCTGTACCTGAACAATTTCAGTGGTCCAATACCAGGCTCGCTGGGGAACC
TATCGAAGCTGCGATTCCTCCGGCTTAACAACAATACCCTGTCTGGTTCAATTCCGCAG
TCTTTGACCAATATTAGCGCACTCCAAGTTCTGGATTTGTCGAACAACAATCTGTCAGG
AGAAGTTCCATCTACTGGCTCGTTTTCACTATTCACCCCCATCAGTTTTGCTAACAATCC
TTTATTGTGTGGTCCGGGTACTACAAAGCCTTGTCCGGGTGCTCCCCCATTCTCCCCAC
CACCTCCATTCAACCCACCAGTACCACCCTCAACACCAGGAAGTAGTGCGTCTAGCAC
TGGAGCAATTGCCGGTGGCGTTGCTGCAGGTGCTGCTCTGCTGTTTGCTGCACCGGCA

ATTGGCTTTGCATGGTGGCGCCGTCGTAAACCACAAGAATATTTCTTTGACGTACCTGC
TGAAGAGGATCCTGAAGTTCATCTCGGTCAGCTTAAAAGATTTTCCCTACGAGAGTTAC
AAGTAGCAACCGATAGTTTCAGCAACAAGAATATTTTGGGCAGAGGTGGGTTTGGCAA
GGTTTATAAAGGACGGCTTGCGGATGGTACATTAGTGGCAGTCAAGAGACTTAAAGAA
GAACGCACACCTGGCGGAGAACTTCAATTTCAGACAGAAGTTGAGATGATCAGCATG
GCCGTGCATCGGAATCTTCTCCGTCTTCGTGGGTTTTGCATGACACCCACCGAACGTCT
GCTTGTCTATCCCTACATGGCTAATGGAAGTGTCGCTTCATGCTTAAGAGAGCGTCCGC
CATCCGAACCTCCACTGGATTGGCTAACTCGAAGACGAATTGCCCTGGGATCAGCCAG
AGGGCTATCTTACTTGCACGACCATTGTGATCCGAAAATTATCCATCGTGATGTCAAAG
CTGCGAATATATTATTGGATGAAGATTTTGAGGCAGTTGTTGGGGATTTCGGCTTAGCCA
AACTTATGGACTACAAGGACACCCATGTTACGACTGCGGTCCGTGGAACAATTGGACA
CATTGCTCCTGAGTACCTTTCCACAGGAAAGTCTTCCGAGAAGACTGATGTCTTTGGAT
ATGGAATTATGCTTCTGGAGCTTATTACAGGACAAAGGGCATTTGACCTTGCTCGGCTT
GCGAATGATGATGATGTCATGTTACTTGATTGGGTGAAAGGACTTTTGAAAGAGAAAA
GGCTAGAAATGCTGGTGGACCCTGACCTCCAGAGCAACTACATAGAAGTCGAGGTGG
AGTCGCTCATCCAGGTCGCCCTGCTCTGCACCCAAGGCTCCCCGACCGAACGTCCGAA
AATGTCCGAAGTGGTCAGAATGCTTGAAGGAGACGGTCTTGCCGAGAGATGGGAAGA
ATG
GCAGAAAGTGGAAGTGGTTCGCCAGGAGGTCGAGATGGCCCCACGCCAGAACTCCGA
ATGGATAGTCGACTCTACCGATAATCTCCATGCCGTCGAGCTATCAGGCCCTAGGTGA
